# Publicly available data sources in sport-related concussion research: a caution for missing data

**DOI:** 10.1186/s40621-024-00484-7

**Published:** 2024-01-30

**Authors:** Abigail C. Bretzin, Bernadette A. D’Alonzo, Elsa R. van der Mei, Jason Gravel, Douglas J. Wiebe

**Affiliations:** 1 Department of Emergency Medicine, Injury Prevention Center, School of Medicine, University of Michigan, Ann Arbor, USA; 2 Department of Epidemiology, School of Public Health, University of Michigan, Ann Arbor, USA; 3 Department of Biostatistics Epidemiology and Informatics, University of Pennsylvania, Philadelphia, USA; 4 Penn Injury Science Center, University of Pennsylvania, Philadelphia, USA; 5 Division of Oncology, Children’s Hospital of Philadelphia, Philadelphia, USA; 6 Department of Criminal Justice, Temple University, Philadelphia, USA

**Keywords:** Concussion, Epidemiology, Electronic health record

## Abstract

**Background:**

Researchers often use publicly available data sources to describe injuries occurring in professional athletes, developing and testing hypotheses regarding athletic-related injury. It is reasonable to question whether publicly available data sources accurately indicate athletic-related injuries resulting from professional sport participation. We compared sport-related concussion (SRC) clinical incidence using data from publicly available sources to a recent publication reporting SRC using electronic health records (EHR) from the National Football League (NFL). We hypothesize publicly available data sources will underrepresent SRC in the NFL. We obtained SRCs reported from two publicly available data sources (NFL.com, pro-football-reference.com) and data reported from the NFL’s published EHR. We computed SRC per 100 unique player signings from 2015–2019 and compared the clinical incidence from publicly available data sources to EHR rates using clinical incidence ratios (CIR) and 95% confidence intervals (CI).

**Findings:**

From 2015–2019, SRC counts from published EHR record data ranged from 135–192 during the regular season, whereas SRC counts ranged from 102–194 and 69–202 depending on the publicly available data source. In NFL.com the SRC clinical incidence was significantly and progressively lower in 2017 (CIR: 0.73, 95% CI: 0.58–0.91), 2018 (CIR: 0.66, 95% CI: 0.50–0.87), and 2019 (CIR: 0.48, 95% CI: 0.35–0.64) relative to the gold-standard EHR. In the pro-football-reference.com data, the documented SRCs in publicly available data sources for other years were ~ 20–30% lower than the gold-standard EHR numbers (CIRs 0.70–0.81).

**Conclusions:**

Publicly available data for SRCs per 100 unique player signings did not match published data from the NFL’s EHR and in several years were significantly lower. Researchers should use caution before using publicly available data sources for injury research.

## Background/context

Across many sports, findings from large epidemiology and surveillance studies continue to inform sport-related concussion (SRC) prevention efforts, including policy and rule changes. American football offers several examples. For example, injury surveillance research identified the protective impact of progressive limitations to collision practices (Bretzin et al. 2022; Pfaller et al. 2019; Broglio et al. 2016). Other research using injury surveillance found that rule changes, including the Ivy League Kickoff Rule (Wiebe et al. 2018) and penalizing targeting (Baker et al. 2021, 2022) were associated with reduced rates SRC, albeit sometimes with unintended consequences (e.g., increasing likelihood of lower extremity injury) (Hanson et al. 2017; Westermann et al. 2016). Such studies demonstrate the importance of valid documentation of incident SRC; if the surveillance data underlying analyses is inaccurate the conclusions may be, too.

Certainly SRC poses a concern in the National Football League (NFL), where SRC accounts for nearly 7% of injuries (Bedard and Wyndham Lawrence 2021). Recent estimates for the likelihood of sustaining of a single SRC in the NFL ranged from 6.2–8.3% per player per season during the 2015–2019 seasons (Mack et al. 2021; Cools et al. 2022), and the likelihood of repeat concussion during the same season ranged from 5.3–8.3% (Cools et al. 2022). Descriptive analyses based on surveillance data identified in-game factors including time during the game, player position, location on the field, level of anticipation, type of play, and helmet location and impact type as important correlates of SRC occurrence in the NFL (Casson et al. 2010; Clark et al. 2017). These findings may help identify players at risk for SRC, and inform rule changes aimed at improving player safety; although, continued investigation of these and other factors is warranted. In parallel, evidence continues to accumulate, but is mixed, regarding negative short- and long-term outcomes of SRC among former NFL players such as worse cognitive function and mood-related (e.g., anxiety and depression) symptoms (Walton et al. 2021; Brett et al. 2021) and neurodegenerative disease (e.g., chronic traumatic encephalopathy) (Mez et al. 2017). As such, American football remains ripe with opportunities for developing and implementing prevention efforts aimed at reducing SRCs.

Notably, accessing high-quality surveillance data from the NFL requires approval through multiple levels of independent review (Dreyer et al. 2019), which represents a potential deterrent for some investigators. This issue may prompt researchers to find novel opportunities using publicly obtainable data sources. For example, injury reports published by the NFL available on NFL.com which reports players’ injuries by week during the season, or pro-football-reference.com which is designed to democratize sports-related data for users to understand and share the sports they love, are obtainable data sources used to test associations between hypothesized risk factors and injury rates. For example, in a recent study Bedard and Wyndham Lawrence (2021) described the epidemiology of injury using the NFL.com injury reports and suggested a decrease in SRC rates between the 2014 to 2015 seasons. Others used these publicly obtainable data sources to separately test the associations between SRC rates and introduction of Playing Rule Article 8 (Baker et al. 2021), unconventional game schedules (i.e., Thursday game day, overseas play) (Teramoto et al. 2017), and the association between in subsequent musculoskeletal injury after sustaining a SRC (Buckley et al. 2022).

Importantly, a recent systematic review identified publicly obtained data sources of SRC in the NFL only captured 70% of medically reported SRC (Inclan et al. 2023a). Another systematic review of studies of the NFL found that publicly obtainable data sources only captured 66% of anterior cruciate ligament (ACL) injuries that were reported by team medical records, the gold standard for documented injury (Inclan et al. 2022). Evidently then, interpreting findings from studies using publicly obtainable data sources warrants caution as incomplete data sources may yield inaccurate results due to measurement bias. Accordingly, we aimed to describe injury counts captured in publicly available data sources and compare injury rates relative to published SRC data originating from the NFL’s electronic health records (EHR). We hypothesize that studies based on publicly available data will underestimate the number of concussions when compared the NFL EHR data.

## Materials and methods

### Data sources

We obtained data using publicly available data sources and previous published aggregate data. Institutional review from the University of Michigan approved this study as exempt; therefore, approval of consent was waived. All methods were performed in accordance with the ethical standards as laid down in the Declaration of Helsinki and its later amendments or comparable ethical standards.

#### Publicly available data sources

Teams are required by the NFL to list all reportable injuries in a practice report, game status reports, and are responsible for reporting in-game injuries (NFL Communications Department 2017). We identified concussions sustained by NFL players across five athletic seasons (2015–2019) using the Pro Football Reference database (pro-football-reference.com) and NFL injury reports (NFL.com). We captured all injuries given the designation “concussion” across the five seasons during the 17 regular season games each year. As this data is only reported weekly and does not give the exact injury date, we determined an incident SRC as the first reported “concussion” for each player. Instances where a player was removed from the injury report for at least one week before reappearing on the report with a concussion were labeled as a subsequent incident concussion. Otherwise, they were considered as ongoing concussions. We also determined the number of players that sustained a concussion if an athlete was given the designation “concussion” at least once during the season. For sensitivity analyses, we additionally captured all injuries designated as “head” using the same methodology. This was completed in R version 4.2.0 (2022-04-22) “Vigorous Calisthenics”.

#### Electronic health records

A recent study on the epidemiology of SRC in the NFL reported that 1,302 concussions occurred among 1,004 NFL players across the 2015–16 through 2019–20 seasons. In this retrospective, observational study Mack et al. (2021) described it is mandated that all NFL player injury data across the 32 clubs requiring medical treatment are reported into the league’s electronic health record (EHR). Due to mandatory injury reporting, standardized training and reporting of injury data, and audits against internal and external sources, we determined these were the best available data to compare against publicly available data sources. In addition, Inclan et al. (2023a) described this data source as the “gold standard” for NFL injury data. Furthermore, these data match an available news release disseminated by the NFL (NFL Player Health and Safety 2015), and additional analyses investigating subsequent or repeat concussions occurring in the same year (Cools et al. 2022). From this data source, we obtained injury counts overall and by time during season (off-season, pre-season, regular season, post-season), and the number of players injured with SRC each year reported in the study. Further, as a measure of exposure, we also obtained the number of unique player signings from this published record.

### Statistical analyses

We used descriptive statistics including frequencies and percentages to describe data from each available data source. After combining data sources, we report the percent of medically documented SRC in the EHR that were accounted for in the publicly available data source(s). We replicate the findings by Mack et al. (2021), in which we computed the clinical incidence of SRC, defined as the number of SRCs divided by the unique player signings per 100 athletes during the regular season (Knowles et al. 2006). Note that our numerator includes multiple concussions across the same player, but our denominator only counts each player once. To compare the clinical incidence between publicly available data and published EHR, we computed clinical incidence ratios (CIR) and 95% confidence intervals (CI). We determined statistical significance if 95% CI excluded one. We performed all analyses in Stata (Stata Statistical Software: Release 17. College Station, TX: StataCorp LLC).

#### Sensitivity analysis

Additionally, we identified novel instances of “head” injury reported in both publicly available data sources during the study period. We did this to account for the possibility that the information available to each of these data sources may lead the curators to classify some SRCs as a head injury in their data rather than as “concussion” specifically. We report the incidence of such injuries annually to assess how including them as suspected SRCs would make the data from public data sources compare to the EHR data gold standard.

## Results

Previously reported EHR data indicated a total of 834 SRC occurring in the regular NFL seasons from 2015–2019. Overall, in the pro-football-reference.com and NFL.com data sources, we identified 694 and 660 incident SRCs across the study period, respectively. Tables 1 and 2 report the SRC data by season and data source. Data from pro-football-reference.com captured 101.0% of SRC in 2015, 81.4% in 2016, 81.1% in 2017, 77.0% in 2018, and 70.3% in 2019 relative to published EHR data. Data from NFL.com captured 105.2% of SRC in 2015, 93.0% in 2016, 72.6% in 2017, 65.9% in 2018, and 47.6% in 2019 relative to published EHR data.

**Table 1:** Description of available sport-related concussion (SRC) data published previously (Mack et al. 2021)

	Mack et al. (2021)						
	Sport-related concussions (SRC) by time of season					Injured players	Unique player signings
	Off	Pre	Regular	Post	Overall		
	*n*	*n*	*n*	*n*	*n*	*n*	*n*
2015	5	83	192	4	284	263	3278
2016	5	71	172	7	255	242	3288
2017	1	92	190	10	302	277	3337
2018	5	80	135	3	223	206	3333
2019	8	79	145	6	238	226	3167

**Table 2:** Description of available sport-related concussion (SRC) data from publicly available data sources

	Pro-football-reference.com		NFL.com	
	Incident SRC	Injured players	Incident SRC	Injured players
	*n*	*n*	*n*	*n*
2015	194	177	202	178
2016	140	124	162	130
2017	154	137	138	133
2018	104	86	89	83
2019	102	98	69	61

**Fig. 1:**
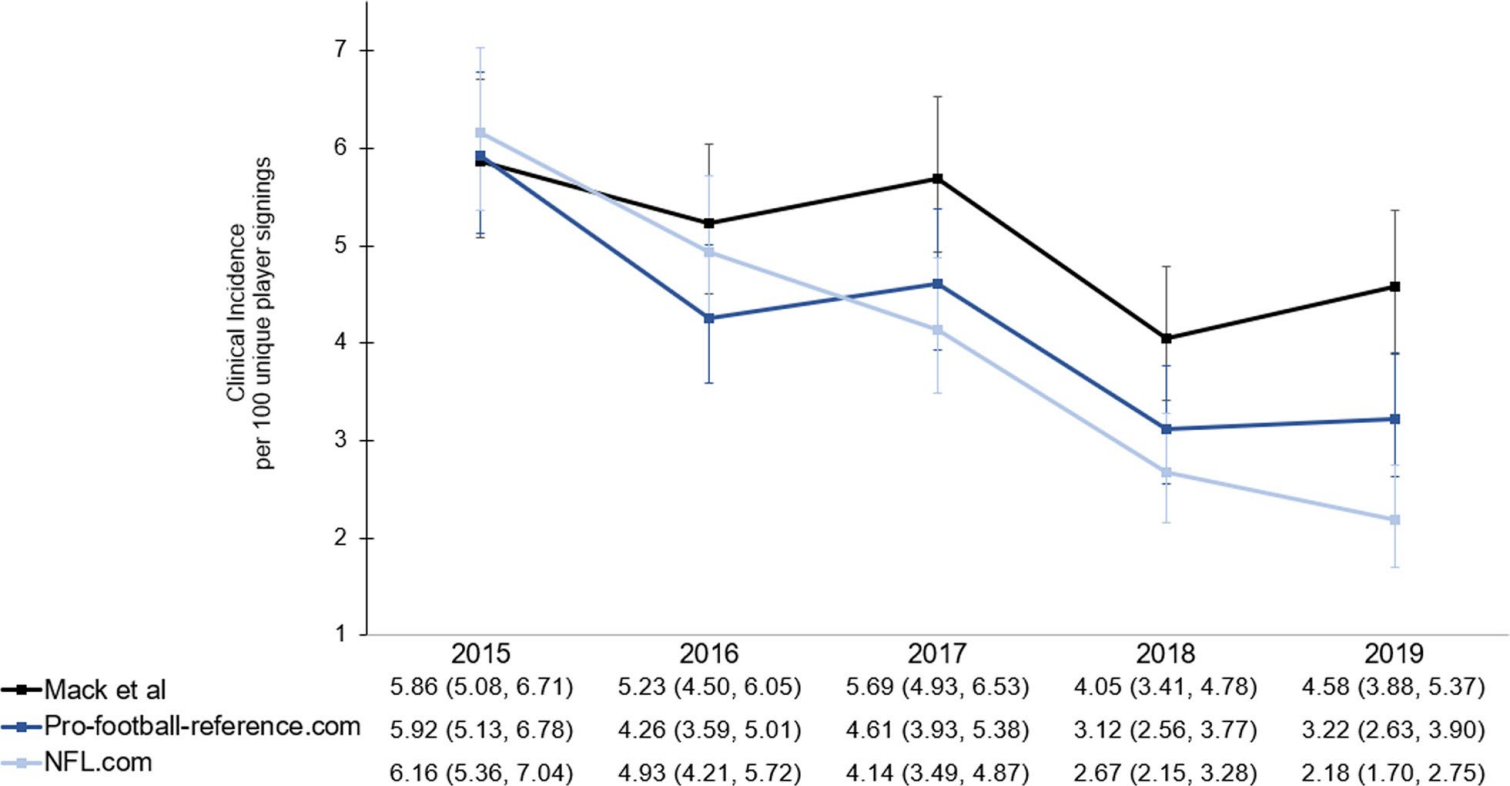
Annual clinical incidence per unique player signings and 95% confidence intervals of sport-related concussion (SRC) reported for a gold-standard source and two sources of publicly available data by year

We report the clinical incidence per 100 athletes by year in Fig. 1. For the published EHR data, annual clinical incidence was 5.86, 5.23, 5.69, 4.05, and 4.58, respectively. The publicly available data from pro-football-reference.com had annual clinical incidences that ranged from 3.12 to 5.92. The publicly available data from NFL.com had similar clinical incidence ranges from 2.18 to 6.16.

**Fig. 2:**
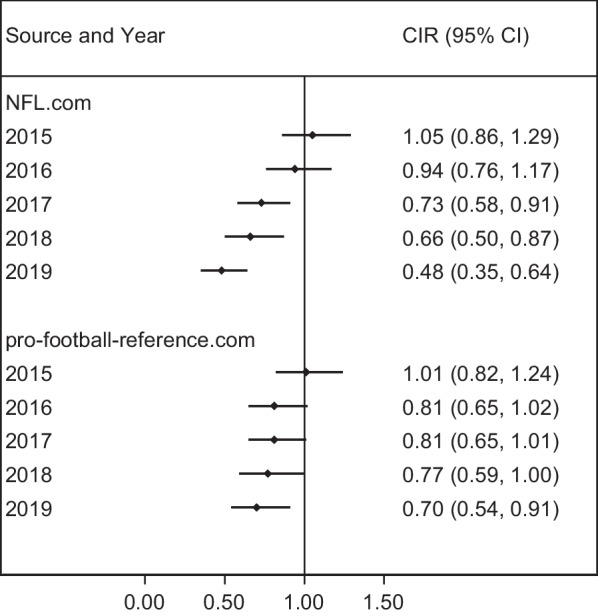
Clinical incidence ratios (CIRs) comparing the annual SRC clinical incidence per 100 unique player signings in two publicly available data sources relative to a gold-standard data source (Mack et al. 2021)

Figure 2 compares annual clinical incidence in each publicly available dataset relative to the clinical incidence in the published EHR data. When comparing pro-football-reference.com to published EHR data, SRC clinical incidence was similar in 2015 (CIR: 1.01, 95% CI: 0.82–1.24) and then were progressively lower annually with an CIR of 0.48 (95% CI: 0.35–0.64) in 2019. When comparing NFL.com to published EHR data, SRC rates were again similar in 2015 (CIR: 1.05, 95% CI: 0.86–1.29) and were progressively lower annually with an CIR of 0.70 (95% CI: 0.54–0.91) in 2019.

The annual number of head injuries, respectively, over the study period as reported by pro-football-reference.com was 7, 8, 0, 3, and 4 and was 4, 3, 0, 2, and 5 as reported by NFL.com.

## Discussion

We used publicly available data sources of injury reports from 2015 through 2019 in the NFL to compare SRC clinical incidence relative to data from the NFL EHR. Our results indicate that publicly available data may underrepresent SRC clinical incidence based on the year and sources of data. For example, pro-football-reference.com and NFL.com only captured 70% and 48% of SRCs in 2019, 77% and 66% in 2018, 81% and 73% in 2017, 81% and 94% in 2016, and 101% and 105% in 2015, respectively, relative to data from published EHR (Mack et al. 2021). Thus based on these results, investigators should question whether publicly available data sources can be used to accurately complete their research. The number of “head” injuries reported in publicly available data sources each year was small—ranging from 0 to 8—relative to the number of concussion injuries reported annually, indicating that misclassified concussions or coding definitions do not account for the differences between the publicly available and gold-standard data.

Our findings, of inconsistencies in medically documented SRC in EHR (Mack et al. 2021) and publicly available data sources, are perhaps unsurprising when considering the findings of recent studies noted above. Specifically, the recent report by Inclan et al. (2023a) that identified that publicly obtainable data sources only accounted for 70% of medically documented SRC in the NFL. Also the systematic review by Inclan et al. (2022) revealed that only 66% of ACL injuries were captured in publicly available data sources. Furthermore, the authors reported that the discrepancies in the percentage of ACL injuries reported in publicly available data sources varied by certain factors (e.g., position, type of play) (Inclan et al. 2022). Therefore, our findings that publicly available data from 2015–2019 only captured 79–83% of medically documented SRC in EHR depending on the source appear to reveal a genuine reason for concern.

The current study provides meaningful findings, considering the prevalence of research reports that disseminate results based on analyses using publicly available data. In fact, a recent systematic review and bibliometric analysis identified an exponential increase in manuscripts using this methodology (Inclan et al. 2023b). One example is a recent research report that identified no increased risk of subsequent musculoskeletal injury after sustaining SRC in NFL players (Buckley et al. 2022), which were in contrast to findings reported at other levels of participation (Lynall et al. 2017; Nusbickel et al. 2022; Herman et al. 2017). Notably, those findings are based on a publicly available data source that only captured less than half of the medically documented SRCs based on the reported descriptives. Therefore, our findings in the current study paired with interpretations of publicly available data by Inclan et al. (2022) and Inclan et al. (2023a) suggest that both data on SRC and musculoskeletal injury reported in prior work may be incomplete or invalid. Another research report found that a change in SRC rate occurred within the study period (Bedard and Wyndham Lawrence 2021); however, based on the reported descriptive statistics, the authors only captured approximately 63% of medically documented SRCs. Moreover, some researchers using publicly available data reported in their discussion that despite well-intentioned rule changes by the NFL the game may not be getting safer (Sheth et al. 2020). Note, working with a partial sample size put the authors at risk for a type-II error. Additionally, if the extent of missing data differed before and after the rule change, that could have produced not only imprecision, but bias as well. Thus, we caution interpretations and policy implications based on publicly available data, due to lower percentages of SRC captured in publicly available data relative to EHR (Mack et al. 2021) found in the current study, and the findings reported by Inclan et al. (2022) and Inclan et al. (2023a).

The above examples are not the only studies using publicly available injury reports to ask data-driven research questions. A recent report evaluated the impact of Playing Rule Article 8—yielding a penalty for a player if they lower the head and use the helmet during a tackle—in the NFL, in which the authors reported decreased SRC rates after enactment of the policy change using publicly available injury reports (Baker et al. 2021). Furthermore, another study reported no significant associations between SRC and playing an unconventional game schedule—that is playing a Thursday game day, or overseas play—that might yield less rest time between games due to a shortened week or travel (Teramoto et al. 2017). However, these findings are based on publicly available injury reports from the PBS Frontline Concussion Watch (http://apps.frontline.org/concussion-watch). Importantly, due to the findings in the current study suggesting differences in SRC rates based on injury reports that are accessible through publicly available websites, it is likely that the data sets utilized in the aforementioned studies may not include all injuries, both SRC and subsequent musculoskeletal injuries (Inclan et al. 2022, 2023a).

There may be many causes for discrepancies between data sources. One such reason may be due to player privacy rights, in which all specific medical information is not released on public domains. Second, there may be competition-related or economic consequences of individual player-level medical records to be publicly accessible. It is also important to note perhaps minor injuries that do not impact practice or game availability do not need to be reported to the public per NFL rules, impacting overall injury data that is publicly available. Therefore, although there are discrepancies in available data sources, we suggest that the inconsistencies are also expected. Still, the discrepancies between data sources found in the current study may indicate either imprecision in a study’s findings if the sample is consistent but small, or bias toward or away from the null hypothesis if missing data are inconsistent across a study period when examining associations in prior work and should be a consideration in future research investigations (e.g., equipment, rules).

### Limitations

This study is not without limitations. The publicly available injury data sources provided few details about each SRC, which aligns with our overall message that research questions that can be pursued with these data sets may be limited. In particular, date of injury was not included, which limits what can be deduced about time out of competition. As an alternative, there was a weekly report indicating a player sustained an SRC, and therefore the player missed out on subsequent play time due to injury. Furthermore, given that the EHR records are not publicly available, we could not access them directly and instead relied on EHR injury counts reported from Mack et al. (2021). Therefore, we could not validate SRC injury counts at the case level in the current study. As noted above, our analyses included injuries designated as “concussion” in the two publicly obtainable data sources, whereas some concussions might be classified as “head” injuries. Our sensitivity analysis indicated this is not an explanation for variability between data sources. Last, it is important to recognize that although EHR data are considered the “gold-standard” source, to date, it may itself have imperfections and may over or underreport SRC which may be due to athlete injury non-disclosure (Jacks et al. 2022; Kerr et al. 2018; Monseau et al. 2021) or even overdiagnosis (Ware and Jha 2015).

## Conclusions

Large surveillance studies play a critical role in enabling researchers to monitor disease and injury trends and identify a need for closer study or prevention efforts (Rothman et al. 2008). In effort to use large data sources that at the surface may seem to be a complete capture, researchers often report having utilized the “most readily available data” including publicly available data sources to answer specific injury-related research questions. This study demonstrates commonly utilized methods for identifying SRC may underestimate the incidence of injury. Specifically, we found publicly obtainable data sources captured a range of 48% to 105% of SRC from the “gold-standard” source depending on the source and year, which represents a critical weakness within the methodology. Therefore, investigators should exercise caution when using publicly available data sources to answer research questions as partial data may yield biased results if the missingness is related to any other variable being investigated, or inappropriate interpretations of results, promoting incomplete findings as stakeholders (e.g., clinicians and policy makers) translate evidence-based findings to clinical practice and policy. Future researchers and journals should understand the limitations associated with use of these sources and seek to use alternative data sources or research strategies.

## Data Availability

This datasets analyzed during the current study are available using injury reports from the pro-football-reference.com and NFL.com.

## Abbreviations

SRC: Sport-related concussion
EHR: Electronic health records
NFL: National Football League
IRR: Injury rate ratios
CI: Confidence intervals
ACL: Anterior cruciate ligament
